# Fibrosarcomatous dermatofibrosarcoma protuberans: a rapidly growing 30 cm mass on the posterior scalp

**DOI:** 10.2478/abm-2023-0060

**Published:** 2023-10-18

**Authors:** Bhoowit Lerttiendamrong, Pavinee Annoppornchai, Pasu Promniyom

**Affiliations:** Division of Plastic and Reconstructive Surgery, Department of Surgery, Faculty of Medicine, Chulalongkorn University, Bangkok 10330, Thailand

**Keywords:** CD34, dermatofibrosarcoma protuberans, fibrosarcomatous transformation, plastic surgery, sarcoma, surgery

## Abstract

Dermatofibrosarcoma protuberans (DFSP) is a rare, slow-growing sarcoma of the skin and subcutaneous tissue, accounting for around 5 cases per million per year. Fibrosarcomatous transformation of DFSP occurs in 10%–15% of DFSP cases, with a higher risk of local recurrence, metastasis, and death. We present a case of a male in his 30s with a complaint of rapidly progressive mass in the occipital region of the head. Within 1 year, the mass enlarged by >30 cm. Physical examination revealed a skin-colored 40×30 cm mass with an overlying skin necrosis at the posterior scalp. Brain, neck, and chest computed tomography (CT) scans were performed. The mass was surgically excised by wide excision with a 2 cm margin. Pathological report confirmed fibrosarcomatous DFSP Grade 3 with decreased CD34 expression. Delayed reconstruction of free flap and split-thickness skin graft were subsequently performed. No recurrence was detected 3 months postoperatively.

Dermatofibrosarcoma protuberans (DFSP) is a rare, slow-growing sarcoma of the skin and subcutaneous tissue, which often affects young and middle-aged adults [1]. The incidence of DFSP is around 0.8–5 cases per million per year, accounting for around 1% of soft tissue sarcoma [2]. Clinical presentation of DFSP is usually a painless, slowly growing, violaceous nodule or plaque [2, 3]. The most common area affected by DFSP is the trunk (42%–72%), followed by the extremities (16%–30%) and the head-and-neck region (10%–16%) [2, 4]. More than 85% of DFSPs are low-grade tumors, while the latter 15% is composed of high-grade fibrosarcomas, with a higher incidence of local relapse and distant metastasis [4].

Fibrosarcomatous transformation of DFSP (DFSP-FS) has been observed in a minority of cases. The transformation is characterized as a more aggressive tumor with a higher rate of recurrence and metastasis [5]. We present a case of a Southeast Asian male with DFSP-FS in the posterior scalp region. Our case provides a unique experience of DFSP-FS as a fast-growing tumor, progressing over 30 cm in a 1-year time frame. We obtained written informed consent in writing from the patient to publish the present case report and associated images.

## Case presentation

**Figure 1.**
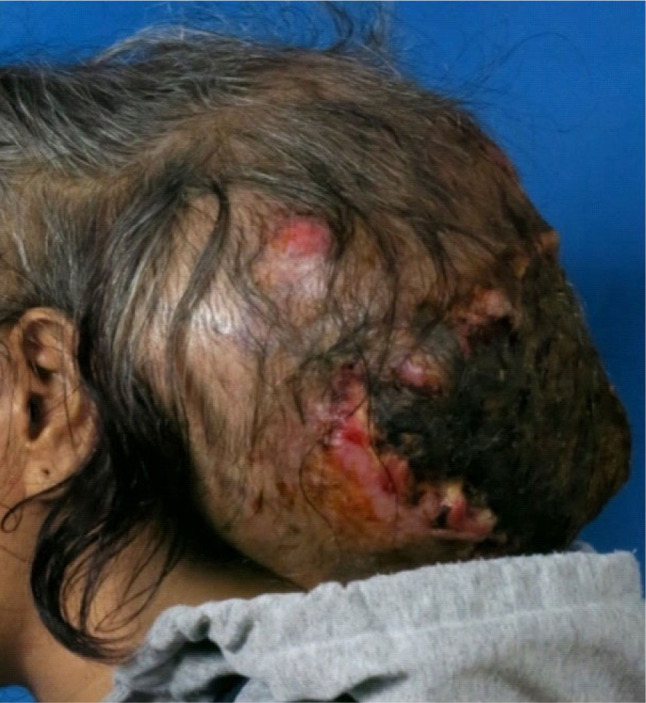
A skin-colored, fixed 40×30 cm mass with an overlying skin necrosis and granulation tissue at the posterior scalp.

A male patient in his 30s presented with a complaint of progressive mass in the occipital region of the head for 1 year. Initially, the mass was around 2 cm in diameter with cystic consistency. The patient denied any pain in the occipital region, without any aggravation from the supine position. Further, 6 months before admission to our center, the mass was progressive in size, measuring around 5 cm in diameter. The patient initially visited a local private hospital, where a computed tomography (CT) scan of the brain was performed. The patient was lost from follow-up at the private hospital due to concerns over the local coronavirus disease-2019 (COVID-19) outbreak. After 3 months since the initial hospital visit, the patient revisited the private hospital with a persisting complaint of progressive mass enlargement. The mass was now disruptive to the patient's daily life, approximating 30 cm in size. The patient denied any pain in the occipital region; however, exacerbation of pain was reported in the supine position or on mass palpation. No fever, significant weight loss, and systemic symptoms were noted. The patient had an underlying condition of Graves disease with large goiter and mild inactive Graves orbitopathy for 2 years, currently receiving propranolol with methimazole with good compliance. He had smoked daily for the past 20 years. He denied any history of alcoholic drinking. No significant family history of cancer was confirmed. On physical examination at our center, a skin-colored, hair-bearing, fixed 40×30 cm mass with an overlying skin necrosis and ulceration at the posterior scalp was found. The mass was soft tissue-like in consistency, with foul-smelling discharge. Bleeding was evident upon contact with the mass. Thyroid enlargement with lid retraction was noted. No other significant physical finding was found. (**Figure 1**).

**Figure 2.**
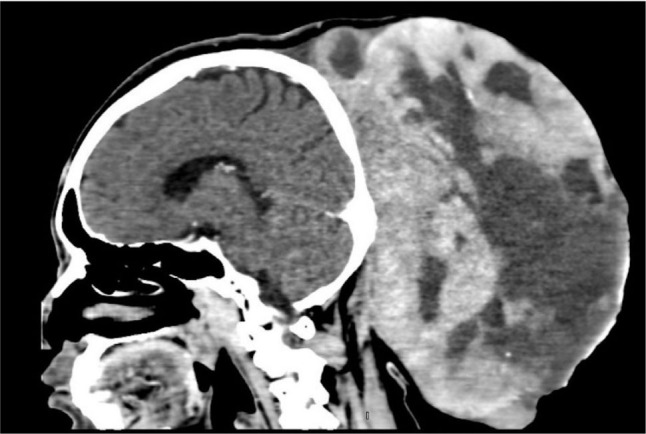
CT scan of brain with contrast (sagittal view).

**Figure 3.**
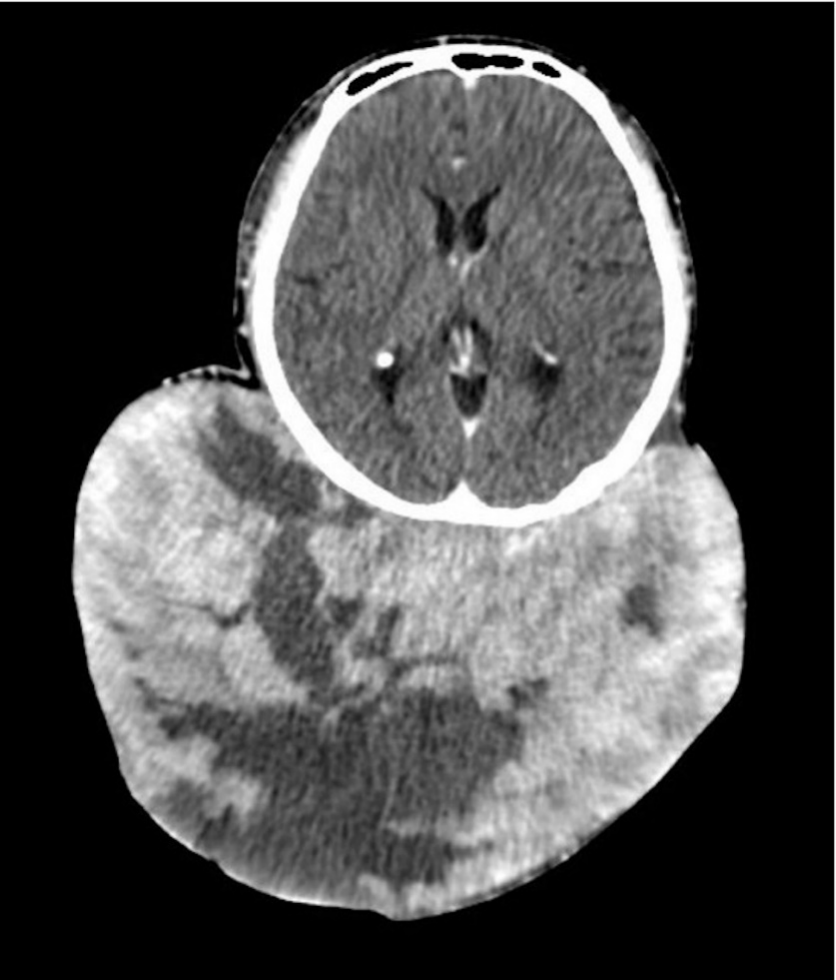
CT scan of brain with contrast (axial view).

Initial brain multidetector CT (MDCT) scan was performed at the private hospital. A 3.4×7.9×8.2 cm oval-shaped, well-defined mass with skin and subcutaneous layer involvement was found in the occipital and upper neck area. Focal patchy enhancing portions of the lower aspect of the mass were noted. No intralesional fat or bone destruction was observed. At the subsequent visit at the private hospital, 3 months after the initial visit, additional brain MDCT scans, as well as neck and chest CT scans, were performed. From the brain MDCT scan, increased mass size was evident (15.4×23.3×22.4 cm). The mass was described as a large, mixed cystic–solid mass at the occipital area and upper neck, with skin and subcutaneous involvement. Patchy enhancing portion at the right lower aspect of the mass and the solid portion was reported (**Figures 2 and 3**). CT scan of the neck revealed huge bilateral enlargement of both thyroid glands. No enlarged lymph nodes in the neck region were reported. No lung nodules, enlarged mediastinal nodes, and liver mass were confirmed from the CT scan of the chest. Mass excision was not performed initially at the private hospital, and the patient was then referred to our center. With regard to the massive mass in the posterior region, no additional magnetic resonance imaging (MRI) scan was performed due to the limitation of the patient's head circumference exceeding the standard MRI bore opening at our center. Surgical wide excision with delayed reconstruction was planned. Administration of potassium iodide and continuation of methimazole were performed preoperatively to control the existing thyroid condition.

**Figure 4.**
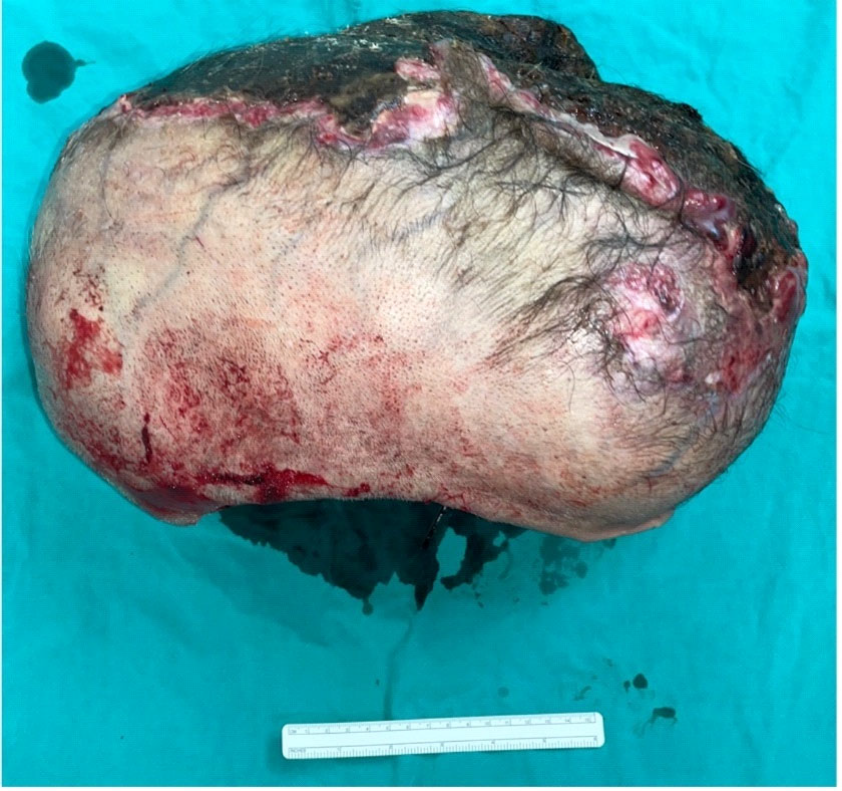
A 38×26.5×19.7 cm subcutaneous posterior scalp mass, compared to a 15 cm ruler.

**Figure 5.**
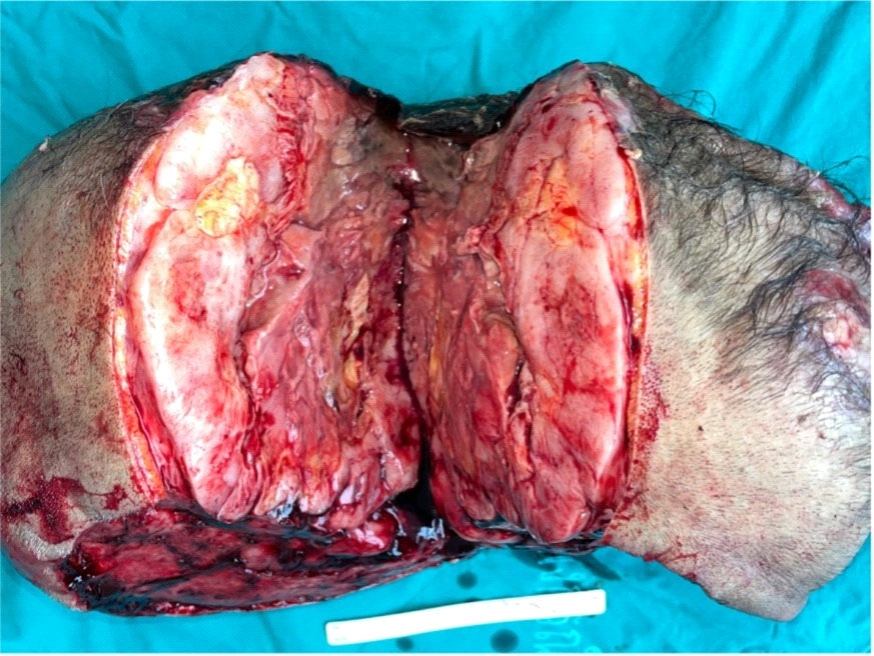
Cross section of the tan white, variegated, lobulated, and rubbery posterior scalp mass.

**Figure 6.**
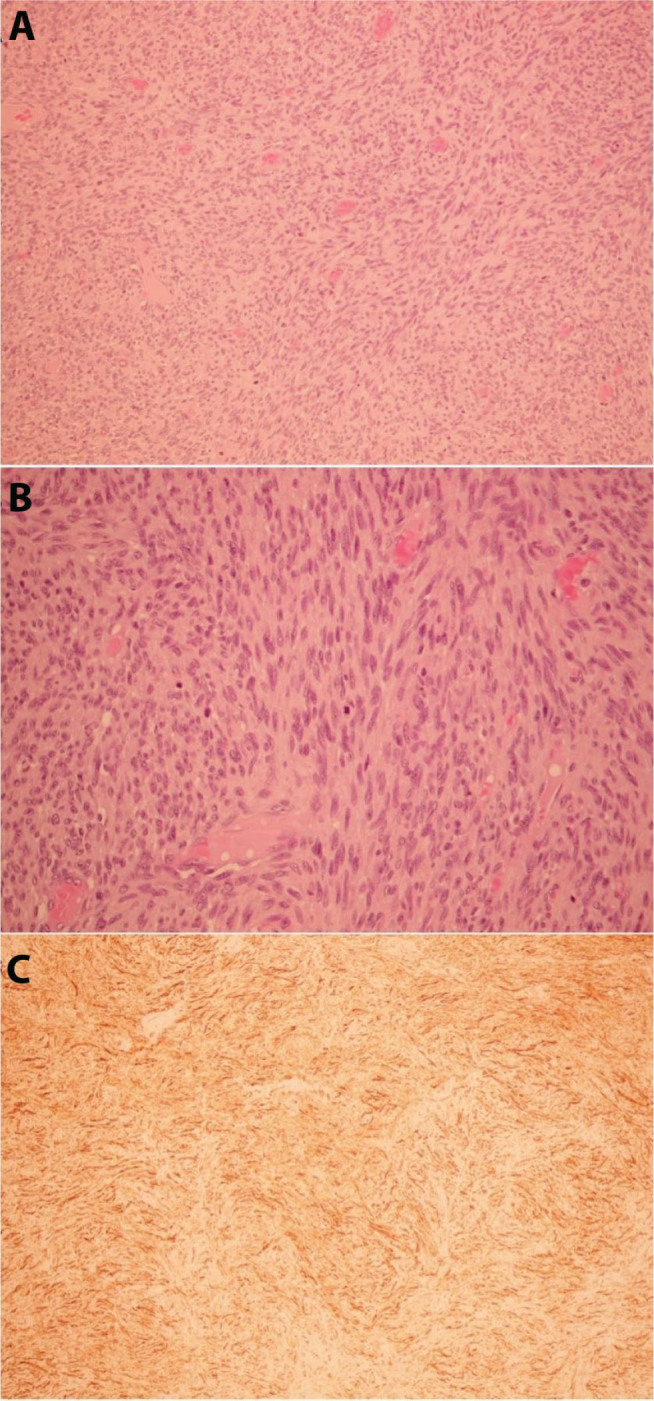
Histopathology and immunohistochemistry of dermatofibrosarcoma protuberans (DFSP) mass on the posterior scalp. **(A)** Hematoxylin-and-eosin-stained images of DFSP showed spindle cell tumor in storiform pattern (200×). **(B)** Hematoxylin-and-eosin-stained images of DFSP showed spindle-shaped cells, arranged in intersecting fascicles that resembled a herring bone appearance (400×). **(C)** Immunostaining profiling displayed strong CD34 expression in the storiform area (200×).

**Figure 7.**
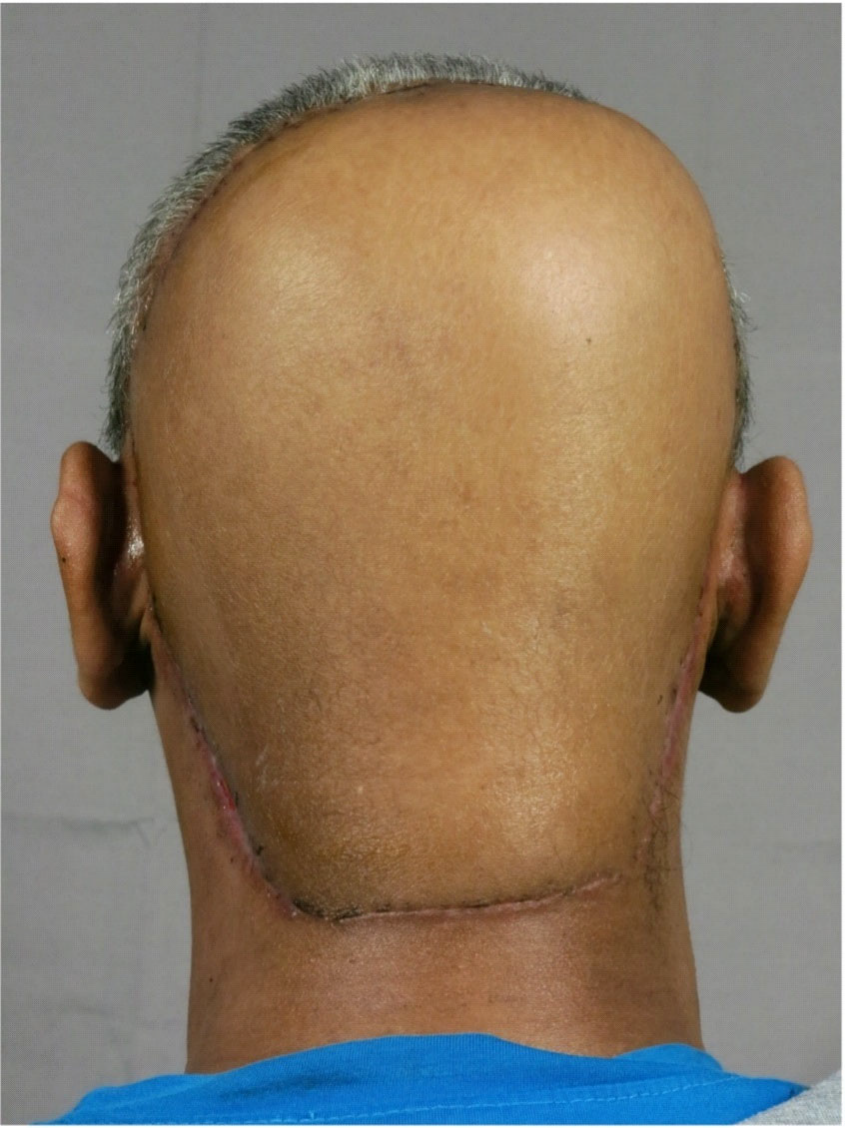
Two months post wide excision of the posterior scalp mass, delayed reconstruction with free flap coverage from the right anterolateral thigh was performed.

We performed wide excision with delayed reconstruction on the occipital mass. A circumferential margin of 2 cm was applied during the wide excision. The subgaleal plane was dissected in the periphery, while the subpericranium was reached centrally. The excised pericranium layer was labeled as the deep margin for the mass. The gross finding of the excised mass was a 38×26.5×19.7 cm subcutaneous mass. Foci of purulent crusts, ranging from 5 cm to 15 cm in the greatest dimension, were identified on the mass surface. Serial sections revealed a tan white, variegated, lobulated, and rubbery mass, with extensive areas of necrotic tissue and pus (accounting for 60% of the total mass area) (**Figures 4 and 5**). Pathological findings confirmed the tissue as fibrosarcomatous dermatofibrosarcoma protuberans (DFSP) Grade 3 (Fédération Nationale des Centres de Lutte Contre le Cancer [FNCLCC] grading system). The tumor involved the epidermis with presence of skin ulceration. All margins were reported as negative for DFSP. No lymphovascular invasion was detected. Immunohistochemical studies of AE1/AE3, EMA, TLE1, S-100, SOX10, H3K27me3, desmin, SMA, MSA, and STAT6 were all reported as negative. CD34 was confirmed as negative in the fascicular part, while the inverse was true for the storiform area (**Figure 6**). Meanwhile, 1 month after the initial mass excision, in order to provide adequate surgical wound healing, delayed reconstruction was performed at the scalp region. Free flap coverage from the right anterolateral thigh (ALT) provided reconstruction. Vascular supplies were anastomosed in an end-to-end fashion between the right ALT free flap pedicle and the superficial temporal vessels. Secondary defect was covered with split-thickness skin graft from the left thigh. Granulation tissue at the defect area in the scalp region was also excised and sent for pathological report. No residual disease was detected from the gross surgical specimen (**Figure 7**).

At the 3-month postoperative follow-up visit since the initial wide excision of the mass, the patient has recovered well without any postoperative complications. No local or systemic tumor recurrence was detected from physical examinations.

## Discussion

Dermatofibrosarcoma protuberans (DFSP) has been reported as a rare, slow-growing cutaneous malignancy [6]. The incidence of DFSP in women is higher than in men, with people 30–50 years old being the most commonly affected age group [3, 6]. DFSP most often arises in the trunk, followed by the upper extremities, lower extremities, and the head and neck region respectively [2, 4, 6]. DFSP in the breast, vulva, penis, and toes has also been reported [7, 8]. No risk factors have been found to be significantly correlated with the development of DFSP [2]. The most common complaint of patient with early DFSP is the presentation of a slow-growing, small, firm, painless, skin-colored mass. Without any initial treatment, the tumor may invade into the fascia, muscle, periosteum, and bone [9]. Distant metastasis to lung, bone, visceral organs, and soft tissues may occur in the advanced stage of DFSP [10]. The progressive mass enlargement usually ranges from several months to years [9]. The mean reported diameter of the mass is between 2 cm and 3.5 cm [9, 11]. Our report presents a case of DFSP in the head and neck region in a male patient in his 30s, which correlated with previous literatures. [3] Notably, our case provides a unique perspective with regard to tumor size and tumor progression. The mass reached a size of >30 cm in its widest dimension, within 1 year since initial detection. The presentation provided a very rare experience for DFSP presentation and diagnosis; however, no distant metastasis was detected.

Fibrosarcomatous dermatofibrosarcoma protuberans (DFSP-FS) is a more aggressive subtype of DFSP, approximating 10%–15% of DFSP cases. The subtype is characterized by intermediate–to-high-grade sarcoma, with more spindle cells, more number of nuclei, and increased mitotic rates compared to DFSP. Immunohistochemistry status also revealed a decreased level of CD34 in DFSP-FS [12]. A systematic review concluded that DFSP-FS has a higher risk of local recurrence (29.8%), metastasis (14.4%), and death from the disease (14.7%), compared to original DFSP. The usual site of DFSP-FS metastasis is the lungs; as such, screening with chest CT scan or MRI in addition to regular physical examinations is advised [13]. In our presenting case of confirmed fibrosarcomatous DFSP, CD34 was reported as negative in the fascicular part, in accordance with previous evidence suggesting the decreased presentation of CD34 in DFSP-FS. At the 3-month follow-up, no local recurrence or distant metastasis was detected on regular physical examination.

Ideal diagnosis of prospective DFSP includes pathologic diagnosis from punch biopsy or excisional biopsy [9]. MRI is useful as a preoperative investigation and surgical planning tool, outlining the tumor's size and extent [14]. CT scan is also beneficial for evaluating distant metastasis; the DFSP mass will appear as a solitary, subcutaneous lobular or nodular architecture with soft tissue attenuation on the CT scan [9, 15]. The National Comprehensive Cancer Network (NCCN) guideline for DFSP 2022 suggested complete skin examination for lesions suspicious for skin cancer. Biopsy and preoperative MRI with contrast were also recommended [16]. In our presenting case, no preoperative MRI was performed due to the inaccessibility of MRI usage as the patient's head circumference exceeded the standard MRI bore. However, brain, neck, and chest CT scans were performed. Brain CT scan demonstrated a well-defined mass with skin and subcutaneous layer involvement. Focal enhancement of the lower aspect of the mass was also reported. No regional or distant metastasis was detected from the scan. Subsequent wide excision was then performed, in accordance with the NCCN recommendations.

Complete excision with negative margins of DFSP lesions has been the primary treatment and mainstay of therapy for the disease [2, 17]. A safe gross margin of 2–3.5 cm from normal tissue to the tumor has been suggested by numerous studies. [2, 18] A reoperation is necessary if the margin is tight or positive [17, 18]. Mohs micrographic surgery has also been proposed as an alternative to wide local excision; however, there is controversy with regard to the definite treatment between Mohs surgery and wide excision [2, 3, 17]. The NCCN guideline 2022 recommended the delay of reconstruction until clear margins are verified, to avoid the risk of tumor translocation in the resection bed [16]. As for definite treatment in the presenting case, our center performed wide excision with a margin of 2 cm. At 1 month postoperatively, delayed reconstruction of ALT free flap was performed to ensure adequate coverage of the excised pericranium layer. The secondary defect was covered with split-thickness skin graft from the left thigh. The granulation area was also excised at the subsequent operation, and no malignancy was detected on all margins. Hair transplantation using body hairs or synthetic biofibers may also be considered as further reconstructive process; however, such intervention was not utilized in this presenting patient [19].

## Conclusion

Our report described a very rare experience of DFSP with fibrosarcomatous differentiation, presenting as a rapidly growing mass. The mass was notable in size, reaching >30 cm in the widest dimension, far exceeding the average mass size for a typical DFSP case. Clinicians should be made aware of DFSP presenting as a fast-growing tumor. We urge clinicians to include DFSP in the differential diagnosis regardless of the progression of mass size.
